# Parliament and the Profession

**Published:** 1917-07-28

**Authors:** 


### Parliament and the Profession.

### National Relief Fund.

Mr. Forster informed Mr. Hogge that the Prince of Wales's Fund had claimed £800,000 from the War Office for separation allowances paid by the Fund, and that the matter having been referred to an arbitrator a sum of £105,000 had been awarded and paid to the Fund.

### Employment of Medical Enemy Prisoners.

Mr. King inquired if alien enemies with full medical qualifications could not be employed in attending their compatriots amongst prisoners, thus liberating British doctors for attendance on British civilians or soldiers. Mr. Macpherson said that he understood that such doctors are employed to a certain extent, but not with very satisfactory results. It is not considered desirable to reorganise the Medical Service as suggested.

### Medical Certificates by à-la-suite Officers.

Mr. Hogge asked the Prime Minister if his attention had been called to the practice of the War Office which forbids *à-la-suite* officers to give a certificate as to the physical condition of any man likely to be brought before a Tribunal; whether this prevents men getting medical certificates from their own doctors; and whether he will take steps to have this injustice removed. Mr. Macpherson, who replied, said that there is no objection to such officers furnishing statements as to the physical condition of individuals for the information of medical boards to assist them in forming an opinion.

### Anti-Typhoid Vaccine.

Mr. Chancellor asked the Under-Secretary of State for War whether the stocks of anti-typhoid vaccine used for the British Army are renewed by the collection of fresh typhoid bacilli; from what source these bacilli are obtained, and whether methods are used to make certain that typhoid and no other bacilli are used in the preparation of the vaccine. Mr. Macpherson replied that the same strain of typhoid bacilli has been used for many years, and fresh typhoid bacilli are not collected for renewal. The vaccine is thoroughly tested by approved methods at every stage in the manufacture to ensure that no other bacilli are present.

